# Declining Time-Trend in Loneliness Levels Among Migrant Children in Urban China, 2006−2019: A Cross-Temporal Meta-Analysis of 40 Studies Published From 2006 to 2022

**DOI:** 10.1155/2024/3094214

**Published:** 2024-09-25

**Authors:** Lin-Feng Ge, Rui-Yao Wu, Bao-Liang Zhong

**Affiliations:** ^1^Department of Psychiatry, Affiliated Wuhan Mental Health Center, Tongji Medical College of Huazhong University of Science and Technology, Wuhan, Hubei, China; ^2^Department of Psychiatry, Wuhan Mental Health Center, Wuhan, Hubei, China

## Abstract

Loneliness has long been a significant psychosocial problem for migrant children in urban China. In recent years, social changes and enhancements in social welfare equity have lessened the disadvantages faced by these migrant children. The current study investigated the time-trend of loneliness levels among migrant children from 2006 to 2019. A literature search was performed within major Chinese- and English-language databases, and studies that reported the means and standard deviations of Children's Loneliness Scale (CLS) scores among Chinese migrant children were included in this cross-temporal meta-analysis. Weighted linear regression was conducted to examine the trend of mean CLS scores over the survey year, and Cohen's *d* value was calculated to assess the magnitude of change. In total, 40 cross-sectional studies conducted between 2006 and 2019 (published by 2022), consisting of 47 cohorts of migrant children and a total sample size of 17,090, were included. Overall, there was a significant downward trend between the survey year and mean CLS score (unstandardized coefficient [*β*] = −0.342, *P*  < 0.001), and Cohen's *d* value of this decline from 2006 to 2019 was 0.411. Similar declining time-trends were also observed among subgroups when broken down by sex, school type, and geographic regions (*β* = −0.182 to −0.589, *P*  < 0.001, *d* = 0.222–0.719). The loneliness levels of migrant children in urban China decreased from 2006 to 2019. Nevertheless, sustained measures and inclusive policies are still needed to mitigate the loneliness levels of Chinese migrant children.

## 1. Introduction

In the 46 years following China's 1978 reform and open policy, the country experienced significant growth in both rural-to-urban and urban-to-urban migration [1–3]. Specifically, from 1982 to 2020, the population of individuals living outside their birth municipality surged from 6.75 million to 375.82 million—a figure that constituted 26.6% of China's total population in 2020 [4]. During this period, the number of rural-to-urban migrant workers dramatically increased from 2 million in 1983 to 295.62 million in 2022 [5, 6]. As many internal migrants relocate with their families, the number of migrant children in China has surged to 71.09 million in 2020 [7]. This signifies that nearly one in every four children under 18 years of age in China is a migrant child.

Similar to international migrants, Chinese individuals relocating to cities for work, study, or residence often confront numerous challenges, such as language barriers, difficulties in assimilating into urban society, disruption in their previous social networks, discrimination, and economic difficulties [8, 9]. In China, due to a unique household registration system called *hukou* that is tied to one's birthplace and a public service resource allocation system based on this *hukou*, migrant children often have limited access to public services in their destination places [10]. This directly results in disparities in healthcare, education, and social welfare access compared to their urban counterparts [11, 12]. As an illustrative example, particularly in earlier years, school-aged migrant children are not eligible to attend public schools with high-quality education and facilities. Instead, the majority could only attend schools specifically designed for migrant children [13, 14]. Unfortunately, these schools often have poor educational quality and facilities and are typically located in urban fringe areas [15]. Due to their marginalized status and social isolation, migrant children in urban China often experience a distinct lack of a sense of belonging to the cities, thereby elevating the risk of feelings of loneliness [16, 17]. Consistent with this, previous studies have reported significantly higher levels of loneliness in migrant children compared to their local urban peers [18, 19].

In recent decades, coinciding with economic growth, the Chinese government has relaxed restrictions, conditionally allowing more migrants to access urban benefits [20, 21]. Education policies have been revised with added emphasis on improving both the quality and equality of education for migrant children [22, 23]. Furthermore, healthcare and social welfare systems have been expanded to more comprehensively cover migrants and their families [24, 25]. As a result of this, we speculate that there would be a long-term downward trend in levels of loneliness among migrant children in urban China.

As social animals, human beings have fundamental needs for social connectedness. Loneliness is “the unpleasant experience that occurs when a person's network of social relations is deficient in some important way, either quantitatively or qualitatively” [26, 27]. Evidence shows that children between the ages of 5 and 7 years already demonstrate a basic understanding of loneliness [28]. Various maladjustment outcomes have been found to be associated with loneliness in children and young adolescents, such as dropping out of school, depressive symptoms, substance abuse, and physical health problems, particularly prominent among those experiencing persistent loneliness [29, 30].

Five theoretical models elucidate childhood loneliness. Evolutionary theory views loneliness as a “social pain” response to exclusion, urging corrective social actions [31]. Psychodynamic theory links loneliness to early mother–child relationship maladjustments, creating an unattainable ideal and pathological loneliness without solid defenses [32]. However, limited empirical support exists for these theories [32]. Social needs theory and cognitive discrepancy theory, prevalent in practice, posit loneliness as arising from unmet social needs, like attachment and integration [33]. Barriers to forming attachments contribute to child loneliness while lacking social integration sustains it. The latter emphasizes subjective perceptions of social contact mismatches [32], stressing individual attributions in shaping children's loneliness.

Contrasting individual-focused theories, interactionist theory suggests loneliness stems from personal, situational, and sociocultural factors [33]. Common personal factors include poverty, introversion, and weak social skills, while situational factors encompass relocation, unemployment, rejection, conflict, and exclusion. For Chinese migrant children, adverse personal and situational factors include low financial status, introversion, migration, peer rejection, unstable relationships, peer discrimination, parental separation, and bullying [34–37].

In China, the prevailing collectivistic culture places importance on relational bonds and group cohesion. Therefore, a key educational objective is to nurture collectivism among children by promoting collaborative work toward shared goals, instilling a sense of group welfare as a priority, and fostering unity and belonging among students [38]. The socially disadvantaged status of migrant children may hinder their integration into peer groups on campus and heighten feelings of alienation, exacerbating their loneliness. With the recent implementation of socially inclusive policies for migrants and their children in China, it is presumed that the social integration levels of migrant children have been on the rise, subsequently reducing their experiences of loneliness.

Investigating the time-trend in loneliness levels among migrant children would deepen our understanding of the effects of social changes on the psychosocial wellbeing of this vulnerable population, as well as inform the development of social welfare policy for this group. However, to the best of our knowledge, there has not been a longitudinal cohort of migrant children followed for more than ten years in China. This makes it difficult to examine the secular trend in loneliness among this population. Fortunately, cross-temporal meta-analysis provides an alternative approach to address this research question if there have been plenty of cross-sectional surveys investigating the prevalence of loneliness in migrant children and carried out in different years over a long period. By using cross-temporal meta-analysis, this study examined the secular trend in loneliness among migrant children in urban China.

## 2. Materials and Methods

The protocol of this cross-temporal meta-analysis was registered in the International Prospective Register of Systematic Reviews (PROSPERO) with the registration number CRD42024533178.

### 2.1. Literature Search

Major Chinese- and English-language databases were searched by using the following keywords: (migrant children OR children of floating people OR children of migrant workers OR children of peasant workers) AND lonel⁣^*∗*^ AND (China OR Chinese). The electronic databases were China National Knowledge Infrastructure, WanfangData, Chongqing VIP, PubMed, PsycINFO, Web of Science, ProQuest, Embase, and Cochrane Library. The search was conducted from the inception of these databases until April 7, 2024.

### 2.2. Inclusion and Exclusion Criteria

For this meta-analysis, we considered eligible those cross-sectional surveys or baseline surveys of cohort studies that used the Chinese version of the Children's Loneliness Scale (CLS) to investigate loneliness levels among migrant children in urban China, reported the sample size and the mean and standard deviation (SD) of the CLS score, and recruited a minimum of 50 children. Because the CLS, originally developed by Asher, Hymel, and Renshaw [39] and validated in its Chinese version by Wang, Jiang, and Ma [40], is the most widely used assessment of loneliness symptoms among children in China, its administration was used as a prerequisite for study inclusion. We excluded studies that involved adults aged 18 years and above, as well as children from kindergartens, because the CLS was not suitable for assessing loneliness in adults and preschool children. If there were two or more reports available from the same study, we included the earliest published one or the one with the most complete data.

### 2.3. Data Extraction

In addition to the sample size, mean, and SD of the CLS score, we also extracted the year when the survey was conducted, the survey site, and the school type of the children (primary vs. middle school students) for each individual study. We also extracted sex- and school-type-specific CLS data if they were available. The survey sites were categorized into east, central, and west regions according to geographic classification criteria in China [41]. If the year of the survey was not specified in the literature, the survey year was assumed to be 2 years prior to the publication year [42].

### 2.4. Statistical Analysis

We used weighted linear regression analysis to establish the regression equation: mean CLS score = *β*⁣^*∗*^ year + intercept. This equation included the mean CLS score as the dependent variable, with the survey year as the only independent variable. The sample size was set as the weight. In this equation, the unstandardized (*β*) and standardized (*r*) coefficients of the year represented the change in the mean CLS score for every one-year increase and the correlation coefficient between the year and mean CLS score, respectively. Using this equation, we computed the average CLS scores for 2006 and 2019. The combined SD of the included studies was calculated according to the formula provided in the Cochrane Handbook, version 5.1.0 [30]. As recommended by Twenge and colleagues, we calculated the magnitude of change in the mean CLS score using the difference in CLS score between 2006 and 2019, divided by the combined SD [42]. The generated quotient equaled Cohen's d, with values of 0.20–0.49, 0.50–0.79, and ≥0.80, denoting small, medium, and large effect sizes, respectively [43–45]. The level of statistical significance was set at *P* < 0.05 (two-sided). We used the SPSS software version 15.0 package (SPSS Inc., Chicago, IL, USA) for all analyses.

## 3. Results and Discussion

### 3.1. Characteristics of Included Studies

**Figure 1:**
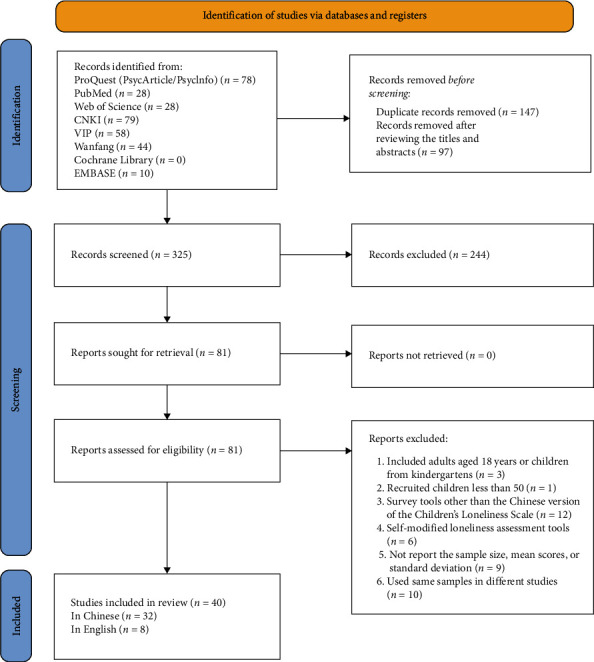
Flowchart of study inclusion for the cross-temporal meta-analysis of loneliness in migrant children in urban China.

**Figure 2:**
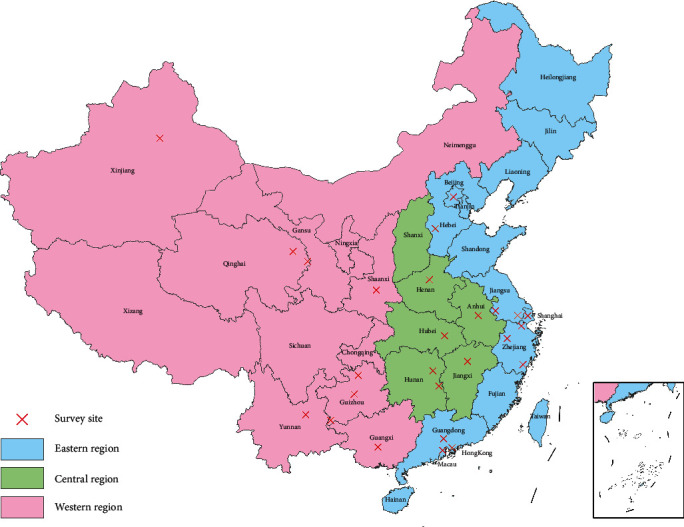
A map showing the survey sites of included studies and the three geographic regions in China.

The literature search identified 313 potential studies. Finally, 40 studies published between 2006 and 2022 met the inclusion criteria and were included [19, 46–84] (Figure 1). These included studies reported the CLS scores of 47 cohorts of migrant children, with a total sample size of 17,090. The survey years ranged from 2006 to 2019.  Figure 2 shows the survey sites of the included studies. The detailed characteristics of the included studies are shown in Table 1.

### 3.2. Time-Trend of Loneliness Levels in Migrant Children

**Figure 3:**
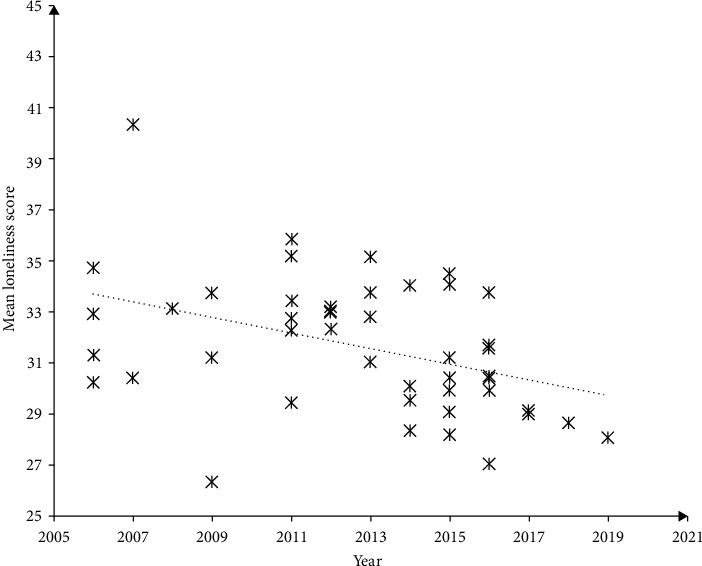
The scatter plot of survey year and mean Children's Loneliness Scale score.

There was a negative correlation between the mean CLS score and the survey year (*r* = −0.507, *P* < 0.001). The corresponding weighted linear regression equation was mean CLS score = −0.342 × year + 719.691, with an *R* square of 0.258 (Figure 3). The unstandardized coefficient was also statistically significant (*P* < 0.001). The magnitude of change from 2006 to 2019, represented by Cohen's *d*, was 0.411. Similar significant declining trends were observed in sex-, school-type-, and geographic-specific mean CLS scores. The largest and smallest magnitudes of change were seen in children from western China (*d* = 0.719) and boys (*d* = 0.222), respectively (Table 2).

## 4. Discussion

The present study addresses an important knowledge gap about the long-term trends in loneliness levels among migrant children amidst the profound sociodemographic changes and rapid urbanization witnessed in China over the past four decades. As hypothesized, we observed a significant downward trend in loneliness among migrant children from 2006 to 2019. This trend was consistent across various subgroups when broken down by sex, school type, and geographic regions, even though the magnitude of change varied.

In addition to policy reforms aimed at reducing social inequity, creating a more socially inclusive environment, and increasing social integration levels for migrant children in cities, we speculate that the increased monthly incomes of these children's migrant parents also play a significant role in lessening their levels of loneliness. For instance, from 2009 to 2022, the average monthly income of migrant workers in China increased from 198 USD to 646 USD [5, 85]. Higher incomes assure financial stability for migrant workers, subsequently improving their children's living conditions and fostering self-esteem and mental wellbeing [86]. Further, recent advancements in accessibility to digital technologies in China, such as social media platforms, the internet, and smartphones, have facilitated greater social connectedness and expanded social networks for migrant children, thereby contributing to reduced levels of loneliness [45, 87, 88]. Importantly, enhanced access to educational opportunities leading to increased peer interactions and improvements in social integration initiatives within migrant communities are likely significant contributors to the observed declining trend.

The varying magnitudes of decline in loneliness levels across different subgroups of migrant children could potentially highlight the heterogeneous impact of social changes on their loneliness. In childhood, boys seem to experience more loneliness than girls [89]. Possibly, since girls are generally more prone to forming close relationships and expressing their feelings compared to boys [90], they might benefit more significantly from the easing of institutional restrictions on migrants in recent years, leading to stronger social connections with urban native children. This could account for the lesser decline in loneliness among boys compared to girls. The observed sex difference in decline in loneliness levels might also be explained by the different patterns of changes in loneliness between boys and girls from childhood to adolescence [32].

The steeper decline in loneliness among migrant children in western China as opposed to eastern China could be attributed to sociocultural differences between the two regions [41]. For example, migrants in eastern China are more often from the central and western provinces, whereas migrants in western China primarily originate from neighboring cities and provinces. As migration-related restriction policies lessen, western migrant children are likely to adapt to and integrate into local society more effectively.

This study has certain limitations. First, the potential for ecological fallacy stemming from the study-level regression analysis is present. Furthermore, given the inherent methodological limitations of cross-temporal meta-analysis—including publication bias, variations in survey methodology, sample characteristics, risk of bias, and quality of reporting—we should be cautious when generalizing these findings. Second, due to a lack of detailed individual-level data, we are unable to decompose age-, period-, and birth cohort-effects from the aggregate-level data. While the observed downward time-trends are likely a result of social changes, we cannot accurately calculate the unique contributions of age-, period-, and birth cohort-factors. Third, cross-temporal meta-analysis can only detect the overall time-trend within a long-term period. Due to this methodological limitation, we are not able to provide detailed data on the fluctuation patterns of loneliness levels during the period from 2006 to 2019. Fourth, as reviewed earlier, loneliness emerges from the intricate interplay of personal, situational, and sociocultural factors. Given that this study is a cross-temporal meta-analysis relying on published studies contrasting with original research rooted in individual data, we are unable to pinpoint the precise factors contributing to the diminishing levels of loneliness among Chinese migrant children. Finally, we did not find any studies about the levels of loneliness in migrant children during the COVID-19 pandemic from 2020 to 2022 in China [91, 92]. It also remains uncertain whether the declining trend of loneliness will persist in the post-pandemic era, given the profound changes the pandemic has instigated worldwide [93].

## 5. Conclusions

In summary, a significant decline in loneliness levels was observed among migrant children in urban China from 2006 to 2019. This trend remained consistent across subgroups defined by sex, school type, and geographic region, likely reflecting the impact of social changes and the enhancements in the socioeconomic status of migrant children in urban China. However, a 2020 comparative study still highlighted significantly higher levels of loneliness in migrant children compared to their nonmigrant counterparts [94]. This indicates that, notwithstanding the long-term downward trend, a marked disparity in loneliness levels persists between migrant and nonmigrant children. Given this finding, coupled with the profound negative effects of the pandemic [95, 96], sustained efforts supplemented by inclusive social policies are crucial to bolster social integration and alleviate the feelings of loneliness among migrant children in China. Considering the high risk of mental health problems in Chinese migrant children, such as depressive symptoms and suicidal and self-injury behaviors, as well as feelings of loneliness [97–99], these children should be prioritized as a key target population for campus-based mental health services in primary and middle schools. Possible services may include mental health education, social skills training, social and emotional support, psychological consultation, periodic monitoring of psychosocial health problems, and, when necessary, psychiatric referral and treatment. To inform the current policy-making for improving the mental wellbeing of migrant children, it is also necessary to conduct more empirical studies to obtain data on the loneliness and other psychosocial health problems during the post-pandemic era.

## Figures and Tables

**Table 1:** Characteristics of included studies for the cross-temporal meta-analysis of loneliness in migrant children in urban China.

Study	Survey site	Survey year	School type of the children	Sampling method	Sample size (boys/girls)	Grade of participants	Mean CLS score (SD)
Peng and Lu [46]	Shenzhen	2006	Primary school	Probability	107 (58/49)	4–6th	32.92 (12.53)
Zhou [49]	Beijing	2006	Primary school	Probability	1438 (737/619)	3 and 5th	33.4084 (10.3792)
Zhang and Xie [48]	Wuhan	2006	Primary school	Convenience	84 (45/39)	3–6th	31.30 (9.25)
Qiu et al. [47]	Nanchang	2007	Middle school	Probability	270 (168/82)	7th	34.8089 (11.1803)
Fan et al. [19]	Beijing	2009	Primary + middle schools	Probability	767 (410/332)	4–9th	31.20 (9.44)
Liu et al. [50]	Hefei	2008	Primary school	Probability	309 (165/144)	4–6th	33.12 (9.37)
Xu and Wang [51]	Hangzhou	2009	Primary school	Probability	148 (92/56)	3and 5th	30.426 (9.256)
Hu 2012 [52]	Wuhan	2011	Primary school	Probability	470 (270/200)	3–6th	32.27 (10.08)
Lin et al. [53]	Nanning	2011	Primary school	Probability	573 (NR)	1–6th	32.74 (11.133)
Yi et al. [54]	Wuhan	2011	Primary school	Probability	239 (NR)	3–6th	32.29 (10.25)
Chen et al. [63]	Beijing	2011	Primary school	Probability	657 (383/260)	4–6th	32.3616 (13.168)
Wang et al. [71]	Xingyi	2012	Primary school	Probability	239 (NR)	5th	33.18 (8.67)
Zhang et al. [38]	Guiyang, Kunming	2012	Primary + middle schools	Probability	336 (192/141)	5–7th	33.03 (10.85)
Zhao [73]	Minhe	2013	Primary school	Convenience	194 (116/78)	3–6th	35.15 (11.666)
Chen et al. [74]	Nanning	2013	Primary school	Probability	347 (187/158)	5–6th	31.04 (10.72)
Geng [55]	Xining	2014	Middle school	Probability	277 (153/124)	7–9th	34.02 (9.5)
Ren [75]	Shanghai	2014	Primary school	Probability	345 (NR)	4-6th	28.35 (10.68)
Zheng [76]	Shijiazhuang	2014	Primary school	Probability	285 (171/114)	5–6th	29.544 (9.0685)
Gong [56]	Shenzhen, Zhongshan, Zhuzhou	2014	Middle school	Probability	486 (281/205)	7–9th	30.08 (10.40)
Li et al. [57]	Nanjing	2015	Primary school	Convenience	401 (227/147)	4–6th	29.07 (10.69)
Mi [77]	Shanghai	2015	Primary school	Probability	601 (361/240)	6th	30.40 (11.84)
Xue [58]	Xi'an	2015	Middle school	Probability	986 (421/310)	7–8th	31.20 (9.60)
Guo et al. [70]	Beijing	2011	Primary school	Probability	426 (269/157)	4–5th	34.40 (11.04)
Shi et al. [78]	Shanghai	2015	Primary school	Probability	311 (186/125)	4–5th	34.4773 (12.0154)
Hu et al. [79]	Beijing	2015	Primary school	Probability	507 (291/216)	4–6th	31.7415 (11.5789)
Luo [80]	Changsha	2015	Middle school	Convenience	233 (NR)	7–8th	28.18 (12.87)
Jiang [81]	Shanghai	2016	Middle school	Probability	307 (180/127)	7–9th	31.68 (11.68)
Wu [82]	Wenzhou	2016	Primary school	Probability	214 (96/118)	3–6th	31.59 (8.285)
Li et al. [83]	Zhengzhou	2016	Middle school	Probability	504 (228/276)	7–9th	30.48 (5.07)
Zhao et al. [59]	Guangzhou	2016	Middle school	Probability	388 (198/190)	7–8th	29.92 (10.24)
Tang et al. [60]	Wenzhou	2016	Primary school	Convenience	450 (NR)	4–6th	33.73 (10.21)
Song et al. [69]	Xinjiang	2016	Primary + middle schools	Probability	411 (211/200)	5–7th	30.42 (12.06)
Ying et al. [67]	Hangzhou, Jiaxing	2016	Primary school	Probability	437 (240/197)	4–5th	27.04 (9.92)
Jiang and Shen [61]	Suzhou	2017	Primary school	Probability	418 (NR)	3–5th	29.00 (12.24)
Chen, et al. 2019 [62]	Shanghai	2017	Primary school	Probability	506 (302/204)	3–6th	29.12 (11.84)
Wang et al. [84]	Guizhou	2018	Primary school	Convenience	324 (171/153)	4–6th	28.64 (11.04)
Kuo et al. [65]	Beijing	2013	Primary school	Probability	837 (495/330)	3–5th	33.76 (11.36)
Chen et al. [68]	Shanghai	2019	Primary school	Probability	335 (198/137)	4–6th	28.0731 (10.9034)
Chen and Yang [64]	Beijing	2012	Primary school	Probability	198 (87/111)	4–7th	32.96 (8.01)
Cao et al. [66]	Beijing	2013	Primary school	Probability	299 (185/114)	4–5th	32.80 (9.76)

**Table 2:** The correlation between survey year and mean Children's Loneliness Scale (CLS) score, regression equation for mean CLS score, and magnitude of change in CLS score from 2006 to 2019.

Subgroup of migrant children	Number of studies	Number of children	Correlation coefficient between year and mean loneliness score (*P*)	Regression equation for mean CLS score	*P* value of the regression coefficient of year	Magnitude of change from 2006 to 2019 (Cohen's *d*)
Overall	40	17,090	−0.507 (<0.001)	−0.342 × year + 719.691	<0.001	0.411
Sex						
Boys	18	3680	−0.276 (<0.001)	−0.182×year + 398.762	<0.001	0.222
Girls	18	2652	−0.413 (<0.001)	−0.304 ^×^year + 642.731	<0.001	0.378
School type						
Primary school	29	11,699	−0.604 (<0.001)	−0.358×year + 752.519	<0.001	0.420
Middle schools or primary + middle schools	11	4965	−0.515 (<0.001)	−0.265×year + 565.609	<0.001	0.339
Geographic region of survey site						
Eastern China	24	10,868	−0.558 (<0.001)	−0.318×year + 672.227	<0.001	0.373
Central China	7	2109	−0.823 (<0.001)	−0.438×year + 913.128	<0.001	0.586
Western China	9	3687	−0.756 (<0.001)	−0.589×year + 1218.398	<0.001	0.719

## Data Availability

The data used to support the findings of this study are included within the article.
